# Fabrication and Performance of Composite Microencapsulated Phase Change Materials with Palmitic Acid Ethyl Ester as Core

**DOI:** 10.3390/polym10070726

**Published:** 2018-07-02

**Authors:** Qing Yin, Zhenguo Zhu, Wei Li, Maolian Guo, Yu Wang, Jianping Wang, Xingxiang Zhang

**Affiliations:** Tianjin Key Laboratory of Advanced Fibers and Energy Storage, School of Material Science and Engineering, Tianjin Polytechnic University, Tianjin 300387, China; yinqing02@163.com (Q.Y.); zhuzhenguotjpu@163.com (Z.Z.); guomaoliansx@163.com (M.G.); wangyu_nankai@163.com (Y.W.); jpwangcn@gmail.com (J.W.)

**Keywords:** phase change material, microcapsules, polyurea, SiO_2_ nanoparticles, polyurethane

## Abstract

Microencapsulation of phase change materials (PCMs) could prevent the leakage of PCMs during solid–liquid phase change process. However, their applications are mainly limited by the compactness and thermal stability of the traditional polyurea shell microcapsules. To increase the thermal compactness and thermal stability of PCM microcapsules, tetraethylorthosilicate (TEOS) was employed to form polymer/SiO_2_ composite shells to enhance the mechanical performance of polyurea and polyurethane microcapsule via interfacial polymerization and in situ polymerization. The morphology and chemical components of the microcapsules were characterized by field-emission scanning electron microscope (FE-SEM) and Fourier transform infrared (FT-IR) spectroscopy, respectively. The thermal properties of the microcapsules were investigated by differential scanning calorimetry (DSC) and thermal gravity analysis (TGA). The results showed the smoothness and compactness of both polyurea–SiO_2_ and polyurethane–SiO_2_ microcapsules enhanced slightly, when compared with that without TEOS addition. Moreover, the SiO_2_ composite shell had good effect on thermal compactness, as the weight loss rate of polyurea–SiO_2_ microcapsules and polyurethane–SiO_2_ microcapsules decreased 3.5% and 4.1%, respectively.

## 1. Introduction

Due to the rapid growth of the consumption of fossil fuels followed by environmental impact and energy resources [1], both scientific societies and industrial communities are concentrating on the improvement of energy utilization efficiency and the development of renewable energy [2,3,4,5,6,7,8]. Phase change materials (PCMs) are different from the conventional thermal energy-storage materials, for their capability in absorbing and releasing latent heat through phase transitions almost without temperature changing [9,10,11,12,13,14,15], and are considered as candidates of renewable energy materials [16,17,18,19,20].

Microencapsulation is a widely-used technique to encapsulate PCMs in the energy storage field. Microencapsulated phase change materials (MicroPCMs) have been studied since the late 1970s, because of their large storage capacity and isothermal nature of the storage process. The main advantages of MicroPCMs are as follows: (1) avoid the leakage of PCMs and isolate PCMs from matrix; (2) increase the heat transfer area of PCMs and improve their performance of thermal conductivity; (3) alter the decentralized state of PCMs and solve the problem that PCMs are incompatible with the surrounding medium in thermodynamics. However, traditional MicroPCMs still have some disadvantages of leakage during the solid–liquid phase transition [21,22], which probably results in some negative effects, such as gradual failure of energy storage, environment pollution and security potential danger. These disadvantages greatly limit practical thermal applications of MicroPCMs [23]. The conventional MicroPCMs with organic polymer shells have been widely studied, which possess good thermal stability but poor in thermal conductivity [24], such as urea formaldehyde resin, melamine formaldehyde resin, poly (methyl methacrylate) and polystyrene [25].

The general preparation methods of microcapsules are classified as physic-mechanical, physic-chemical, and chemical methods. The microcapsules prepared via physic-mechanical method generally have large particle size, rough surface, and low thermal storage capacity. In addition, microcapsules prepared via physic-mechanical method could obtain heat capacities of as high as about 145.2 J·g^−1^. However, it would lead to great difficulty with the method for processing at scale. The chemical methods, especially in situ polymerization or interfacial polymerization, are widely used for the preparation of microcapsules. In situ polymerization of amino resins, such as melamine formaldehyde (MF) resin and urea formaldehyde (UF) resin, are usually selected as the shell of MicroPCMs [25,26]. However, this method generally results in formaldehyde residue in microcapsules shells inevitably, so polyurethane and polyurea are two optimally used polymers to fabricate MicroPCMs [26]. The polyurea shell is usually prepared by aromatic isocyanates and amine, such as diphenylmethanediisocyanate (MDI), toluene diisocyanate (TDI) [27], but aromatic isocyanates can hydrolyze into highly toxic phenylamine. Aliphatic isophoronediisocyanate (IPDI) as monomer to replace aromatic isocyanates can form polyurea microcapsules with good yellowing resistance and without any phenylamine [28]. In addition, during the fabrication of polyurea, MicroPCMs, the toxic cosolvent tetrahydrofuran, acetone, and cyclohexane are usually chosen to form the miscibility of *n*-alkane PCMs and isocyanate. To solve these issues, ethyl palmitate has been used, and avoids the malodor in traditional MicroPCMs. In the end, certain defects exist in the thermal stability and compactness of traditional polyurea MicroPCMs.

In this paper, both polyethylene glycol 400 (PEG 400) and tetraethylorthosilicate (TEOS) were selected as functional shell-forming monomers to enhance the thermal stability and compactness of traditional polyurea MicroPCMs. PEG 400 has a macromolecule flexible chain, which can improve the flexible and resilient of polymer, while TEOS carry out hydrolysis polymerization to form polyurea–SiO_2_ or polyurethane–SiO_2_ composite shell. Therefore, it is anticipated that, the compactness and thermal stability would be enhanced via incorporating SiO_2_ into organic polyurea or polyurethane shell.

## 2. Experimental Section

### 2.1. Materials

Ethyl palmitate (99 wt %) was purchased from Sinopharm Chemical Reagent Co., Ltd. (Beijing, China). Polyethylene glycol 400 (PEG400) and tetraethylorthosilicate (TEOS, 99 wt %) were purchased from Aladdin (Shanghai, China). Isophoronediisocyanate (IPDI, 99 wt %) and diethylenetriamine (DETA, 99 wt %) were obtained from Adamas Reagent Co., Ltd (Shanghai, China). Styrene–maleic anhydride copolymer solution (SMA) employed as surfactant was kindly supplied by Institute of Functional Fiber, Tianjin Polytechnic University (Tianjin, China).

### 2.2. Microencapsulation Process

#### 2.2.1. Fabrication of SiO_2_–Polyurea Microcapsules

The microcapsules were synthesized by the interfacial polymerization: SMA (5 g) was dissolved into deionized water and formed the aqueous phase. Ethyl palmitate (15 g), IPDI (7 g) and TEOS (2 g) were mixed and stirred at 30 °C for 15 min to form a homogeneous oil phase. Then, the oil phase was mixed with aqueous phase by a high-shear dispersion homogenizer at the speed of 5000 rpm for 10 min to form stable emulsion in a beaker. After that, DETA (7 g) was slowly dropped into the emulsion at a stirring rate of 500 rpm for 2 h at 40 °C. Then, the temperature was raised to 70 °C rapidly, and then, the emulsion was cured under 500 rpm of agitation for 2 h to form polyurethane–SiO_2_ composite microcapsule slurry.

#### 2.2.2. Preparation of Polyurea–Polyurethane–SiO_2_ Microcapsules

The microcapsules were synthesized by the interfacial polymerization. Two different phases were prepared, PEG-400 (3 g) and SMA (5 g) were dissolved into deionized water, and formed the aqueous phase. Ethyl palmitate (15 g), IPDI (7 g), and TEOS (2 g) were mixed and stirred at 30 °C for 15 min to form a homogeneous oil phase, then the oil phase was mixed with aqueous phase by a high-shear dispersion homogenizer at the speed of 5000 rpm for 10 min to form an emulsion in a beaker. After, DETA (4 g) was slowly dropped into the emulsion at a stirring rate of 500 rpm for 2 h with a mechanical agitation, while the temperature of the solution was controlled at 40 °C, then the temperature was raised to 70 °C rapidly, and the emulsion was cured under 500 rpm of agitation for 2 h to form a polyurea–polyurethane–SiO_2_ MicroPCMs slurry. After being washed by hot distilled water, the prepared MicroPCMs were then treated by filtration and drying.

### 2.3. Characterization

#### 2.3.1. Fourier Transform Infrared Spectroscopy (FT-IR)

FT-IR spectrum, recorded on a FT-IR Uecior 22 spectrophotometer (Shanghai, China), was used to identify the structure of the microcapsules. Samples were prepared as KBr pellets, and scanned against a blank KBr pellet background at wave numbers ranging from 4000 to 500 cm^−1^ with a resolution of 4.0 cm^−1^.

#### 2.3.2. Field Emission-Scanning Electron Microscopy (FE-SEM)

A scanning electron microscope (SEM, Hitachi S-4800, Tokyo, Japan) was used to observe the morphology and structures of these composite microcapsules. The samples were prepared by dropping an aliquot (20 µL) of microcapsules onto a slide; subsequently, the slide was mounted on metal stubs using conductive tape and vacuum-coated with a thin layer of platinum using a sputter coater.

#### 2.3.3. The Phase Change Properties Analysis

The phase change properties were characterized using a differential scanning calorimeter (DSC, NETZSCH 200 F3, Bavaria, Germany) in the range of −20–80 °C. A specimen of 5~10 mg was encapsulated in an aluminum pan under a nitrogen atmosphere and first heated from −20 to 80 °C at a rate of 10 °C/min and kept at 80 °C for 2 min. Subsequently, the specimen was cooled to −30 °C at a rate 10 °C/min and kept at that temperature for 2 min. Finally, the specimen was heated again from −20 to 80 °C with a rate of 10 °C/min. DSC thermograms in the first cooling and the second heating process were recorded.

#### 2.3.4. Thermal Gravimetric Analysis (TGA)

The thermal stabilities of the samples were investigated using the thermogravimetric analyzer (TGA, Netzsch, STA409PC, Bavaria, Germany) in the range of 40–550 °C with a heating rate of 10 °C/min under a nitrogen atmosphere.

### 2.4. Compactness Test

The prepared microcapsules (10.00 g) were packed in conical filter membrane and stored at 120 °C for 1 h. Quality of microcapsules is measured every ten minutes, and then the change of the weight loss rates could be examined.

## 3. Results and Discussion

### 3.1. Morphology and Inner Microstructure of Various MicroPCMs

**Figure 1:**
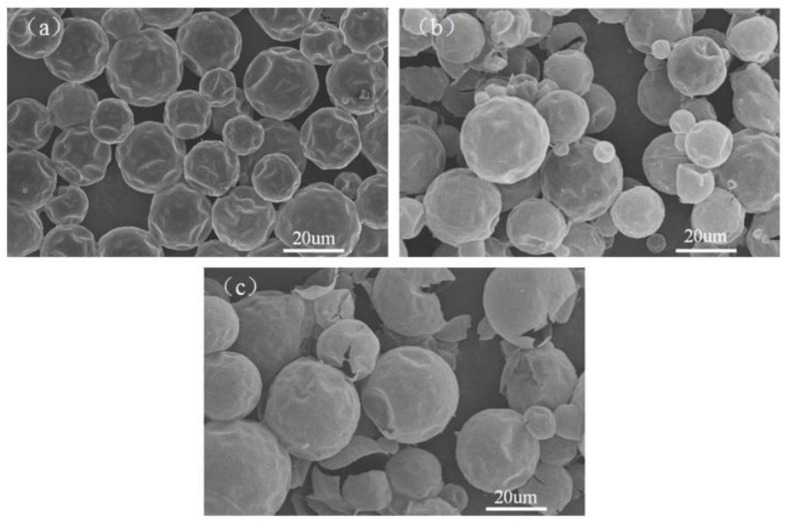
Effects of various core/shell ratios on morphology of polyurethane MicroPCMs: (**a**) 1:1; (**b**) 2:1; and (**c**) 3:1.

Figure 1 presented the effects of various core/shell ratios on morphology of polyurethane MicroPCMs. During SEM sample preparation, we used a glass plate to apply certain force onto the capsules after they were adhered onto the conductive carbon tape. When the core/shell ratio was 1:1 (a), the spherical microcapsules were integrated, and the smooth surfaces were compact, however, there were some dents on the surface. When the ratio increased to 2:1 (b), few cracking phenomena appeared on the surface of capsule, and more cracking phenomena appeared when core/shell ratio enhanced to 3:1. The exact value of shell strength cannot be obtained, however, the strength of various capsules could be simply compared. The strength mechanical performance and stability decreased with shell thickness turned thicker and thicker accordingly.

**Figure 2:**
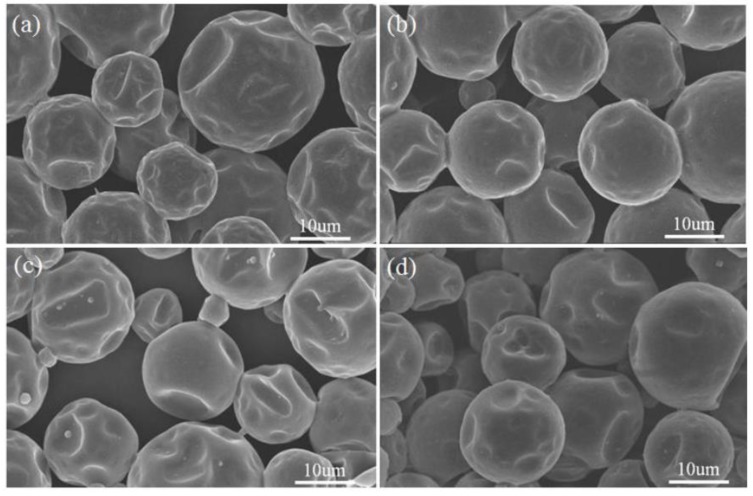
SEM micrographs of various MicroPCMs: (**a**) polyurea microcapsules; (**b**) polyurethane microcapsules; (**c**) polyurea–SiO_2_ microcapsules; (**d**) polyurethane–SiO_2_ microcapsules.

SEM images of all MicroPCMs were presented in Figure 2. The shape of the polyurea microcapsules was spherical, and the surface was seriously dented. While the polyurethane microcapsules were regular spherical with relatively smooth surfaces, the dents have been greatly improved, and there was only a small degree of denting. The reason was probably due partly to the fact that the stiffness and mechanical performance of polyurethane shell synthesized between IPDI and DETA was enhanced, compared with that of polyurea shell containing PEG soft segments, which is prone to depress or deform when the microcapsule is subjected to external forces or collision of particles. By contrast, the polyurethane shell composed of high crosslinked amide bonds possessed high strength mechanical performance, and could withstand the outer force. Compared with polyurea microcapsules, the surface depression of hybrid polyurea–SiO_2_ microcapsules have turned out slightly better, this was mainly because the polyurea shell material was formed already, before the SiO_2_ protective layer was formed inside, caused by relatively slower hydrolysis polymerization. The protective inner SiO_2_ layer almost had no influence on the surface of polyurea shell, but played a certain supporting role. The shape of polyurethane–SiO_2_ microcapsules were more spherical and the surfaces are smooth. When the TEOS hydrolysis occurred, the polymerization shrinkage forced on polyurethane shell increased, and resulted in the dent appearance on the polyurethane–SiO_2_ shell.

**Figure 3:**
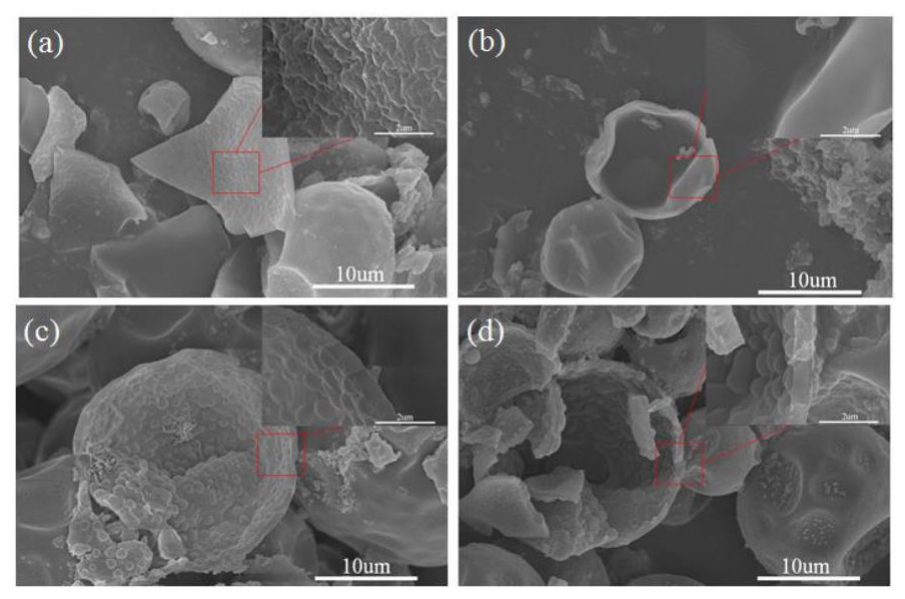
SEM micrographs of inner shell of various MicroPCMs: (**a**) polyurea microcapsules; (**b**) polyurethane microcapsules; (**c**) polyurea–SiO_2_ microcapsules; (**d**) polyurethane–SiO_2_ microcapsules.

As presented in Figure 3, it can be seen from the inner shell surfaces of various microcapsules that the inner surface of the polyurea microcapsules was rough, as shown in Figure 3a. Relatively speaking, the inner surface of the polyurethane microcapsules became much more smooth in Figure 3b. Many hemispherical convex parts appeared on the inner shell of both (c) polyurea–SiO_2_ microcapsules and (d) polyurethane–SiO_2_ microcapsules, which were possibly a result of hydrolysis polymerization of TEOS. The bulging of the inner surface indicated that the silica formed was composed with the polyurethane from the tetraethoxysilane.

**Figure 4:**
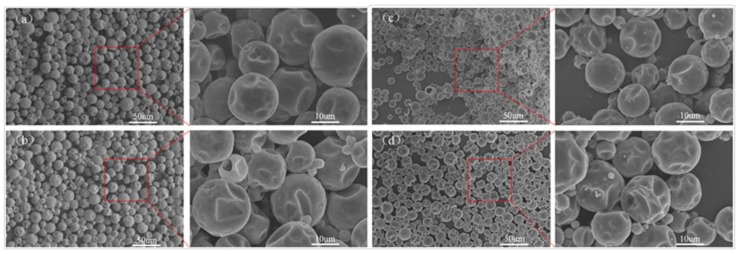
Influence of various addition amounts of TEOS on morphology of polyurethane MicroPCMs: (**a**) 0 g; (**b**) 2 g; (**c**) 4 g; and (**d**) 6 g.

Figure 4 showed the influence of various addition amounts of TEOS on morphology of polyurethane MicroPCMs. The spherical microcapsules with smooth and compact surface had integrity without TEOS addition. With the increase of addition amounts of TEOS, the degree of concave on the shell became a little more significant, this phenomenon may be attributed to that some TEOS did not polymerized by hydrolysis completely, or the resultant SiO_2_ products did not form a continuous enhanced layer inside the shell.

### 3.2. FT-IR Spectroscopy Analysis of Microcapsules

**Figure 5:**
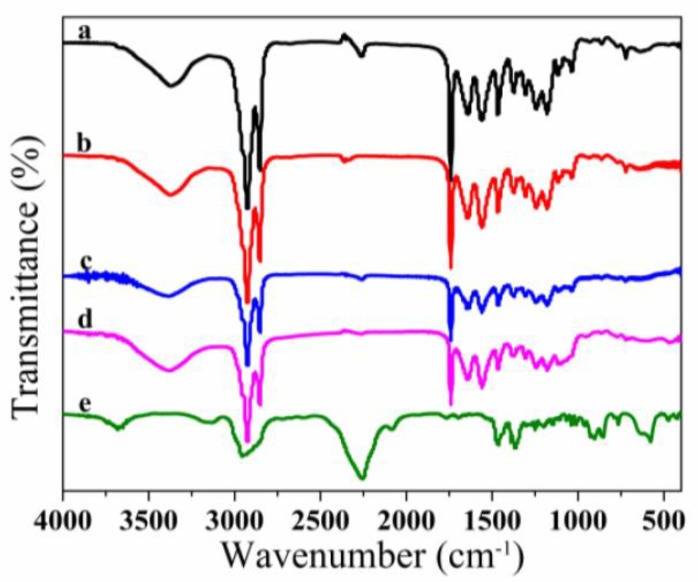
FT-IR spectra of various MicroPCMs and shell-forming monomer: (**a**) polyurea microcapsules; (**b**) polyurethane microcapsules; (**c**) polyurea–SiO_2_ microcapsules; (**d**) polyurethane–SiO_2_ microcapsules; (**e**) IPDI monomer.

The FT-IR spectra of four kind MicroPCMs were preseted and compared in Figure 5. An absorption band at 2256 cm^−1^ belonged to the isocyanate group (–NCO) in spectra (e) (Figure 5). As monomer IPDI was polymerized to form polyurethane or polyurea polymer, the absorption band at 2256 cm^−1^ disappeared in spectra (a), (b), (c), and (d) (Figure 5), indicating that there was no isocyanate residue left in the microcapsules shell. There were several reasons for this phenomenon. Firstly, the isocyanate group of the IPDI reacted fast with the amine group of the DETA. Secondly, DETA was compatible with the ethyl palmitate, so it could diffuse into the interface of the emulsion and ensured the –NCO in IPDI was completely reacted. The peaks at 2925 and 2845 cm^−1^ in spectra (a) were respectively assigned to methylene and methyne C–H in the ethyl palmitategroup. The peak at 1735 cm^−1^ in spectrum (a) was assigned to the C=O in ethyl palmitate group. The peaks at 1250 cm^−1^ in spectra (b) were assigned to C–O–C in the polyurethane polymer chain. The results indicated that the polyurethane were probably polymerized. The FT-IR spectra of MicroPCMs (c) and (d) containing SiO_2_ were compared in Figure 5. Both of the spectra (c) and (d) possessed almost identical absorption band at 1084 cm^−1^, which were respectively assigned to C–O–C. These results indicated that the SiO_2_ had been polymerized through TEOS hydrolysis and polymerization reaction.

### 3.3. EDS Energy Spectra Analysis

**Figure 6:**
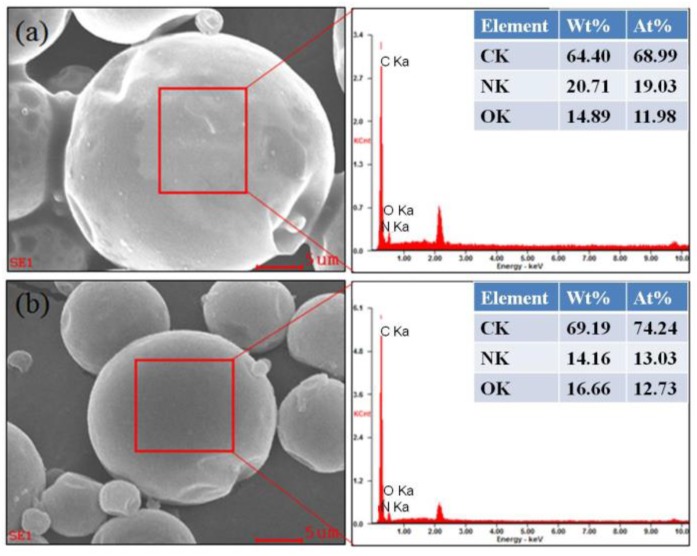
The EDS of MicroPCMs: (**a**) polyurea microcapsules; (**b**) polyurethane microcapsules.

**Figure 7:**
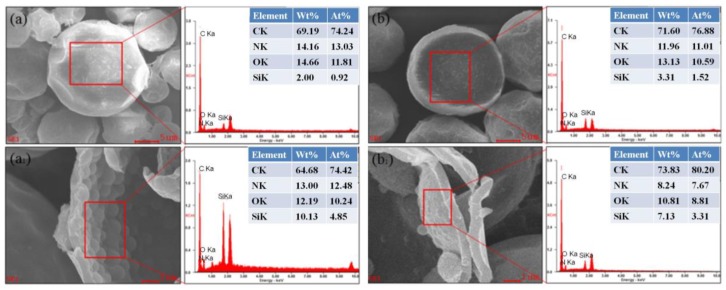
The EDS of (**a**) and (**a_1_**) polyurea–SiO_2_ microcapsules; (**b**) and (**b_1_**) polyurethane–SiO_2_ microcapsules.

From Figure 6a, it can be found the element C, N, and O of polyurea microcapsules were 64.40, 20.71 and 14.89 wt %, respectively. Accordingly, the element C, N, and O of polyurethane microcapsule were 69.19, 14.16, and 16.66 wt %, respectively. Figure 7a,a_1_,b,b_1_ showed the EDS data of polyurea–SiO_2_ microcapsules and polyurethane–SiO_2_ microcapsules. The polyurea–SiO_2_ or polyurethane–SiO_2_ MicroPCMs composites were consisted of ethyl palmitate cores, SiO_2_, and polyurea or polyurethane, which contained silicon (Si), carbon (C), oxygen (O), hydrogen (H), and nitrogen (N). The silicon (Si) weight contents of outer and inner shell of polyurea–SiO_2_ capsule were measured as 2.00 wt % and 10.13 wt %, respectively. While the silicon (Si) weight contents of outer and inner shell of polyurethane–SiO_2_ composite capsule were 3.31 wt % and 7.13 wt %, respectively. As can be seen from the EDS spectra of the inner and outer surfaces of both polyurea–SiO_2_ microcapsules and polyurethane–SiO_2_ microcapsules, the content of silicon contained on the inner surface was obviously higher than that of the silicon contained on the outer surface, which further illustrated that the hydrolysis polymerization mainly occurred inside the shell rather than other position of shell. The Si characteristic peaks and corresponding data indicated that the SiO_2_ was possibly formed on the inner shell by the hydrolysis of TEOS, and the microscale semisphere Si composites could also be clearly seen from the Figure 7a_1_,b_1_.

### 3.4. Compactness Analysis

**Figure 8:**
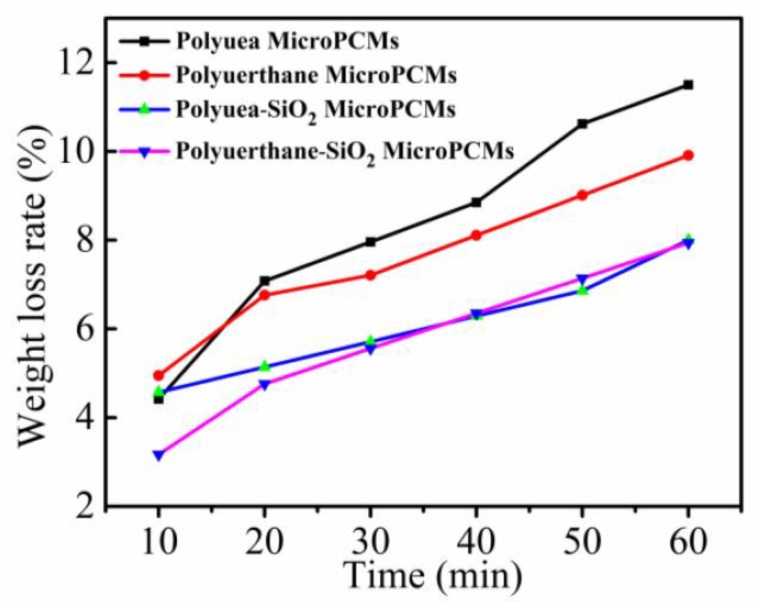
Weight loss rates curve comparison of various MicroPCMs.

The weight loss rates of various MicroPCMs were shown and compared in Figure 8. The weight of the microcapsules decreased with increasing heat-treatment time in Figure 8. The weight loss rate of polyurea microcapsules was approximately 11.5%, while for polyurethane microcapsules, it was about 9.9%. Compared with polyurea microcapsules, the compactness of polyurethane microcapsules were little enhanced, indicating that there was some effect on improving the compactness of MicroPCMs with reduced denting. By contrast, the weight loss rates of polyurea–SiO_2_ microcapsules and polyurethane–SiO_2_ microcapsules were around 8.0% and 7.9%, respectively. The mass loss rate of both polyurea–SiO_2_ and polyurethane–SiO_2_ microcapsules was significantly reduced, indicating that SiO_2_ composition shell formed by the hydrolysis polymerization of TEOS, deceased the porosity of microcapsules and enhanced the compactness of microcapsules.

### 3.5. Melting and Crystallization Behaviors of MicroPCMs

**Figure 9:**
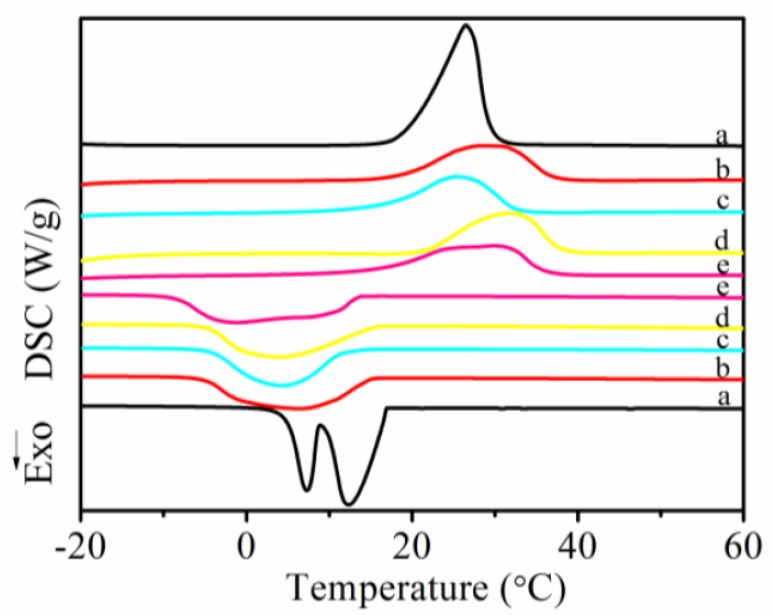
DSC thermograms comparison of various MicroPCMs and pure ethyl palmitate: (**a**) ethyl palmitate; (**b**) polyurea MicroPCMs; (**c**) polyurethane MicroPCMs; (**d**) polyurea–SiO_2_ MicroPCMs (**e**) polyurethane–SiO_2_ MicroPCMs.

The DSC curves of various MicroPCMs and pure ethylpalmitate were shown in Figure 9. Various MicroPCMs had single endothermic peak at range of 25.5–31.6 °C. While two peaks from high to low temperature were observed on the DSC cooling curve of pure ethyl palmitate attributed to the formation of different crystal form or transformation of crystal type, and the latent heat was 203 J/g approximately. Interestingly, sole peak from high to low temperature could be observed on the DSC cooling curve of MicroPCMs. The reason was that the shell of MicroPCMs had a microscale space restriction effect and heat insulation effect on ethyl palmitate core. The encapsulation ratios could be calculated using

$$\eta \%=\frac{∆H_{\mathrm{MicroPCMs}}}{∆H\times \frac{M_{c}}{M_{\mathrm{MicroPCMs}}}}\times 100$$

where Δ*H*_MicroPCMs_ represented the melting enthalpy of MicroPCMs, and Δ*H*_c_ (203 J/g) represented the melting enthalpy of ethyl palmitate as measured by the DSC. *M*_MicroPCMs_ and *M*_c_ were the mass of the microcapsules raw materials and bulk ethyl palmitate, respectively. The encapsulation ratio of all MicroPCMs exceed in 100%, by calculation, indicating that the shell contents of MicroPCMs were less than theoretical values. There was a few main reasons accounting for that the polymerization reaction of shell between shell-forming monomers, such as IPDI, DETA or PEG 400, could not react completely. Moreover, the phase change enthalpies on exothermic peak and endothermic peak of polyurethane-SiO_2_ microcapsules are 126.1 J.g^−1^ and 122.1 J.g^−1^, and the PCM content of MicroPCMs can be calculated by the ratio of ethyl palmitate of MicroPCMs to that of corresponding ethyl palmitate bulk. The average enthalpy of polyurethane-SiO_2_ microcapsules is as high as 124.1 J.g^−1^, and the measured PCM content can be calculated as 61.0 wt%. Observing the DSC cooling curve of MicroPCMs, the supercooling appears in all MicroPCMs samples (Table 1) to some extent. The supercooling was probably caused by decreasing nucleating agents in microscopic space and confined crystallizaion, and the crystallization temperature reduced with the decrease of particle diameter. Due to the reducing crystallization temperature, the latent heat was released at a lower temperature and in a wider temperature range.

### 3.6. Thermal Stability of MicroPCMs

**Figure 10:**
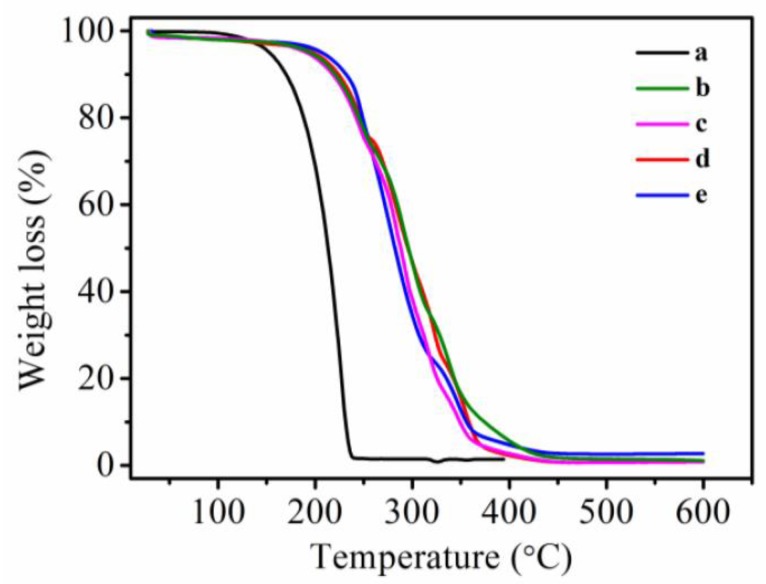
Thermal gravity analysis (TGA) curve comparison of various MicroPCMs and pure ethyl palmitate: (**a**) ethyl palmitate; (**b**) polyurea microcapsules; (**c**) polyurethane microcapsules; (**d**) polyurea–SiO_2_ microcapsules; (**e**) polyurethane–SiO_2_ microcapsules.

Thermal stability was considered as an important performance of encapsulated PCMs for their practical application. The thermal stabilities of ethyl palmitate, polyurea MicroPCMs, polyurethane MicroPCMs, polyurea–SiO_2_ and polyurethane–SiO_2_ MicroPCMs were evaluated by means of TGA, as shown in Figure 10. The weight of ethyl palmitate, polyurea MicroPCMs, polyurethane MicroPCMs, polyurea–SiO_2_ and polyurethane–SiO_2_ MicroPCMs decreased with increasing temperature. Pure ethyl palmitate began to evaporate and lose weight at approximately 195.1 °C, and lost weight completely at approximately 236.2 °C. The thermal resistant temperature of polyurea MicroPCMs was about 217.7 °C, indicating that the ethyl palmitate encapsulated in shells probably decomposed, and evaporated at a higher temperature, owing to the protection of the polyurea microcapsule shell. The thermal resistant temperature of polyurethane MicroPCMs was around 215.6 °C, which was similar with that of polyurea MicroPCMs. While the thermal resistant temperature of MicroPCMs with SiO_2_–polyurea composite shell was 213.4 °C, there was no enhancement in thermal stability, indicating that there was no effect on the compactness and thermal stability of shell by incorporation of SiO_2_. However, the thermal stability of MicroPCMs with polyurethane–SiO_2_ composite shell was slightly increased to around 233.0 °C from 215.6 °C of polyurethane MicroPCMs, indicating more compact and stable organic–inorganic microstructure was likely formed in the polyurethane–SiO_2_ composite shell.

## 4. Conclusions

In the current study, we synthesized stable phase change microcapsule by emulsion polymerization and interfacial polymerization method. FE-SEM micrographs showed the optimal core/shell ratios of polyurethane MicroPCMs was 1:1, and the suitable addition of TEOS was 2 g. The smoothness and compactness of both polyurea–SiO_2_ and polyurethane–SiO_2_ microcapsules enhanced slightly when compared with that without TEOS. The melting enthalpy of polyurea–SiO_2_ microcapsules and polyurethane–SiO_2_ were 120.1 and 121.1 J·g^−1^, while the crystallization enthalpy of polyurea–SiO_2_ microcapsules and polyurethane–SiO_2_ were 122.1 and 126.1 J·g^−1^, respectively. The SiO_2_ composite shell had good effect on thermal compactness, and the weight loss rate of polyurea–SiO_2_ microcapsules and polyurethane–SiO_2_ microcapsules decreased 3.5% and 4.1%, respectively. The TGA results also showed that polyurethane–SiO_2_ microcapsules decreased 16 °C, indicating composites shell could improve thermal stability of the polyurea microcapsules.

## Figures and Tables

**Table 1:** Phase change performance comparison of various MicroPCMs and pure ethyl palmitate.

Sample	*T*_mo_^a^ (°C)	*T*_mp_^b^ (°C)	*T*_me_^c^ (°C)	Δ*H*_m_ (J·g^−1^)	*T*_co_^d^ (°C)	*T*_cp_^e^ (°C)	*T*_ce_^f^ (°C)	Δ*H*_c_ (J·g^−1^)	Δ*H*_i_^g^ (J·g^−1^)	PCM content ^h^ (wt%)
a	19.6	26.5	29.3	203.9	16.9	12.3	8.8	202.7	203.3	—
b	18.7	28.5	36.9	123.4	15.0	6.2	−5.0	128.6	126.0	62.0
c	16.9	25.5	32.4	110.5	11.5	4.2	−4.1	113.7	112.1	55.1
d	21.9	31.6	37.5	120.1	15.3	3.7	−4.4	121.0	120.5	59.3
e	16.5	29.8	35.8	122.1	13.3	−1.2	−8.3	126.1	124.1	61.0

(a) ethyl palmitate; (b) polyurea microcapsules; (c) polyurethane microcapsules; (d) polyurea–SiO_2_ microcapsules; (e) polyurethane–SiO_2_ microcapsules. Note: ^a^ onset temperature on DSC heating curve; ^b^ peak temperature on DSC heating curve; ^c^ enthalpy on DSC heating curve; ^d^ onset temperature on DSC cooling curve; ^e^ peak temperature on DSC cooling curve; ^f^ enthalpy on DSC cooling curve; ^g^ average enthalpy of |Δ*H*_m_| and |Δ*H*_c_|; ^h^ ratio of Δ*H* of MicroPCMs to that of corresponding ethyl palmitate bulk.
